# Effect of Normobaric Hypoxia on Alterations in Redox Homeostasis, Nitrosative Stress, Inflammation, and Lysosomal Function following Acute Physical Exercise

**DOI:** 10.1155/2022/4048543

**Published:** 2022-02-25

**Authors:** Mateusz Maciejczyk, Anna Zalewska, Małgorzata Gryciuk, Katarzyna Hodun, Miłosz Czuba, Kamila Płoszczyca, Małgorzata Charmas, Jerzy Sadowski, Marcin Baranowski

**Affiliations:** ^1^Department of Hygiene, Epidemiology and Ergonomics, Medical University of Bialystok, 2C Adama Mickiewicza Street, 15-022 Bialystok, Poland; ^2^Department of Restorative Dentistry and Experimental Dentistry Laboratory, Medical University of Bialystok, 24A Marii Sklodowskiej-Curie Street, 15-276 Bialystok, Poland; ^3^Students Scientific Club “Biochemistry of Civilization Diseases” at the Department of Hygiene, Epidemiology and Ergonomics, Medical University of Bialystok, 2c Mickiewicza Street, 15-233 Bialystok, Poland; ^4^Department of Physiology, Medical University of Bialystok, 2C Adama Mickiewicza Street, 15-022 Bialystok, Poland; ^5^Faculty of Rehabilitation, Józef Piłsudski University of Physical Education in Warsaw, Marymoncka 34, 00-968 Warsaw, Poland; ^6^Department of Kinesiology, Institute of Sport-National Research Institute, Trylogii 2, 01-982 Warsaw, Poland; ^7^Department of Biochemistry and Physiology, Faculty of Physical Education and Sport in Biała Podlaska, Józef Piłsudski University of Physical Education in Warsaw, Akademicka 2, 21-500 Biała Podlaska, Poland

## Abstract

Hypoxia is a recognized inducer of oxidative stress during prolonged physical activity. Nevertheless, previous studies have not systematically examined the effects of normoxia and hypoxia during acute physical exercise. The study is aimed at evaluating the relationship between enzymatic and nonenzymatic antioxidant barrier, total antioxidant/oxidant status, oxidative and nitrosative damage, inflammation, and lysosomal function in different acute exercise protocols under normoxia and hypoxia. Fifteen competitive athletes were recruited for the study. They were subjected to two types of acute cycling exercise with different intensities and durations: graded exercise until exhaustion (GE) and simulated 30 km individual time trial (TT). Both exercise protocols were performed under normoxic and hypoxic (FiO_2_ = 16.5%) conditions. The number of subjects was determined based on our previous experiment, assuming the test power = 0.8 and *α* = 0.05. We demonstrated enhanced enzymatic antioxidant systems during hypoxic exercise (GE: ↑ catalase (CAT), ↑ superoxide dismutase; TT: ↑ CAT) with a concomitant decrease in plasma reduced glutathione. In athletes exercising in hypoxia, redox status was shifted in favor of oxidation reactions (GE: ↑ total oxidant status, ↓ redox ratio), leading to increased oxidation/nitration of proteins (GE: ↑ advanced oxidation protein products (AOPP), ↑ ischemia-modified albumin, ↑ 3-nitrotyrosine, ↑ S-nitrosothiols; TT: ↑ AOPP) and lipids (GE: ↑ malondialdehyde). Concentrations of nitric oxide and its metabolites (peroxynitrite) were significantly higher in the plasma of hypoxic exercisers with an associated increase in inflammatory mediators (GE: ↑ myeloperoxidase, ↑ tumor necrosis factor-alpha) and lysosomal exoglycosidase activity (GE: ↑ N-acetyl-*β*-hexosaminidase, ↑ *β*-glucuronidase). Our study indicates that even a single intensive exercise session disrupts the antioxidant barrier and leads to increased oxidative and nitrosative damage at the systemic level. High-intensity exercise until exhaustion (GE) alters redox homeostasis more than the less intense exercise (TT, near the anaerobic threshold) of longer duration (20.2 ± 1.9 min vs. 61.1 ± 5.4 min—normoxia; 18.0 ± 1.9 min vs. 63.7 ± 3.0 min—hypoxia), while hypoxia significantly exacerbates oxidative stress, inflammation, and lysosomal dysfunction in athletic subjects.

## 1. Introduction

An inevitable consequence of functioning under aerobic conditions is the production of reactive oxygen (ROS) and nitrogen species (RNS). ROS and RNS are typical by-products of oxygen metabolism and important messengers in cellular signal processing. Under physiological conditions, ROS and RNS are involved in energy metabolism, erythropoiesis, muscle contraction, and other biochemical processes [1–3]. The signaling activity of free radicals is based on the modulation of several transcription factors, e.g., NF-*κ*B (nuclear factor kappa-light-chain-enhancer of activated B cells) and HIF-1 (hypoxia-inducible factor-1), which results in S-nitrosylation of proteins and induction of second-order transmitter formation, as well as changes in cellular redox status [4–6]. However, ROS and RNS overproduction and/or insufficient antioxidant defense can cause redox imbalance, leading to cellular damage by oxidation and nitration. Such a state is defined as oxidative and nitrosative stress, which plays an essential role in many contemporary diseases, including metabolic [7, 8], neurodegenerative [9, 10], autoimmune [11, 12], and neoplastic disorders [13, 14]. Interestingly, factors inducing oxidative and nitrosative stress involve physical exercise [2, 15–17]. It was shown that overproduction of ROS/RNS occurs both during and after training [2, 15, 16]. A direct source of free radicals is the activity of mitochondrial enzymes and membrane oxidases (e.g., NADPH oxidase (NOX)), disturbances of ion homeostasis (especially iron and calcium ions), or changes in lysosomal function. The rate of ROS formation depends on the intensity and duration of exercise, the degree of training of the subjects, their age, sex, and diet [2, 15, 16, 18, 19]. It was shown that regular long-term aerobic exercise, especially at high intensity, is responsible for a significant increase in oxidative stress through intensified oxygen consumption [19–21]. Lipid peroxidation of muscle cells results in decreased fluidity and higher permeability of cellular membranes and enhanced oxidation of proteins and their tissue aggregation, as well as ROS-mediated DNA injury, causing an inflammatory response, delayed muscle soreness, and the release of intramuscular enzymes into the blood [15, 22, 23]. Nevertheless, little is known on redox homeostasis, nitrosative stress, and inflammatory response after acute physical intervention.

Nowadays, altitude/hypoxic training is becoming increasingly popular in sports [24–27]. Exposure to hypoxia leads to stimulation of HIF-1, which, apart from regulation of erythropoiesis and angiogenesis, is also a regulator of activity of glycolytic enzymes, mainly phosphofructokinase (PFK-1) [28, 29]. Therefore, improvements in aerobic and anaerobic capacity may occur. However, hypoxia and subsequent reoxygenation are also responsible for ROS/RNS overproduction, during prolonged exposure to altitude, as well as during intermittent hypoxic training [29–31]. This is caused by disruption of the mitochondrial respiratory chain, disturbances in arachidonic acid metabolism, or migration/activation of immune cells during regular physical activity [24, 25, 30, 31]. Nevertheless, there is a lack of studies evaluating the relationship between antioxidant systems, oxidative and nitrosative cell damage, inflammation, and lysosomal function under normoxic and hypoxic conditions. We speculate that even acute physical exercise can induce oxidative stress and inflammation, and hypoxia can exacerbate these disorders. As interest in high-altitude sports grows, it is essential to understand the differences in redox homeostasis between various protocols of acute physical intervention. Previous studies have examined only a few aspects of redox homeostasis [30, 32, 33] and ultimately have not systematically studied the effects of normoxia and hypoxia during acute exercise. Therefore, the present study is aimed at evaluating the relationship between (1) enzymatic and nonenzymatic antioxidant barrier, (2) total antioxidant/oxidant status, (3) oxidative and (4) nitrosative cell damage, (5) biomarkers of inflammation, and (6) lysosomal function in different protocols (different intensity and duration) performed under normoxic and hypoxic conditions.

## 2. Materials and Methods

### 2.1. Participants

The investigation conformed with the principles outlined in the Declaration of Helsinki and was approved by the Bioethics Committee of the Medical University of Bialystok (approval no. R-I-002/325/2019). All subjects gave their informed consent before their inclusion in the study.

Fifteen well-trained male competitive athletes (12 cyclists and 3 triathlonists) aged 25.4 ± 8.4 years, with BMI of 21.6 ± 1.8 kg/m^2^, body fat content of 9.2 ± 2.1%, and VO_2_max of 61.4 ± 3.1 mL/kg/min were recruited for the study. Their average training experience was 6.3 ± 2.0 years. Only candidates with a valid medical certificate confirming the absence of contraindications to the practice of competitive sport activity were accepted.

### 2.2. Experimental Design

The subjects were tested on two occasions, separated by 14 days, in normoxic and hypoxic (FiO_2_ = 16.5%, equivalent to 2,000 m asl) conditions applied in random order. The tests were performed in a laboratory room equipped with a normobaric hypoxia system (AirZone 25, Air Sport, Poland) allowing to freely manipulate the oxygen concentration in the room air. Temperature (19°C), humidity (50%), and CO_2_ concentration (700–800 ppm) were controlled and held constant. The study participants were blinded to exercise conditions. The athletes were instructed to maintain their regular diet and supplementation throughout the experiment and avoid caffeine intake for 24 h preceding each test. All participants arrived at the camp one day before the start of each test series and consumed the same meals throughout their stay (40 kcal/kg/d, 50% carbohydrates, 20% proteins, and 30% fats).

On the first day of each stay, two hours after a light breakfast, the subjects performed graded cycling exercise beginning with a workload of 120 W, which was subsequently increased by 40 W every 3 minutes until volitional exhaustion. The total duration of exposure to hypoxia during this test was ~35 min. On the second day, following 24 h of rest and two hours after a light breakfast, the athletes performed a simulated 30 km individual time trial (TT) in a mountainous terrain. The TT was preceded by a 15 min warm-up, carried out according to the individual preferences of the athletes, under the same oxygen concentration as during the main exercise. The total duration of exposure to hypoxia during this test was ~90 min. Both tests were performed on subjects' personal bicycles connected to an electromagnetic bicycle trainer (Cyclus 2, RBM Elektronik-Automation GmbH, Leipzig, Germany). During each test series, the athletes were allowed to consume water ad libitum. The oxygen saturation of arterial blood (SpO_2_) and heart rate (HR) was measured using the WristOx2 pulse oximeter (Nonin Medical Inc., Plymouth, USA).

### 2.3. Blood Collection

Blood samples were taken from the antecubital vein into 4 mL EDTA tubes at three-time points: before the exercise, immediately after its completion, and following 30 min of rest. They were kept on ice until centrifugation at 375 × g for 10 min at 4°C. Platelet-rich plasma was transferred to a fresh plastic tube, and the leukocyte-rich buffy coat was thoroughly removed. Separated erythrocytes were suspended in ice-cold PBS and centrifuged at 800 × g for 10 min, and the upper layer and the remaining buffy coat were discarded. Red blood cells were then resuspended in PBS and flash-frozen in liquid nitrogen. Platelet-rich plasma was centrifuged at 2000 × g for 10 min to sediment platelets. The supernatant was then transferred to a fresh plastic tube and recentrifuged at 5000 × g for 10 minutes to obtain platelet-free plasma. All samples were stored at -80°C until analysis.

### 2.4. Biochemical Assays

All reagents were of analytical grade and purchased (unless otherwise stated) from Sigma-Aldrich (Nümbrecht, Germany, or Saint Louis, MO, USA). Redox determinations were performed in duplicate assays: assessment of antioxidant enzymes in erythrocyte samples and assessment of nonenzymatic antioxidants, oxidative and nitrosative stress products, inflammatory mediators, and lysosomal enzymes in the plasma samples. The absorbance and fluorescence were determined using an Infinite M200 PRO multimode microplate reader (Tecan Group Ltd., Männedorf, Switzerland). The results were then standardized to 1 mg of total protein content, as reported in several other publications [34–40]. The total protein level was evaluated with the bicinchoninic acid (BCA) method, using a commercial kit (Thermo Scientific PIERCE BCA Protein Assay (Rockford, IL, USA)), with bovine serum albumin (BSA) as a standard. Redox determinations were performed no more than two months after the samples were frozen.

### 2.5. Enzymatic Antioxidant Barrier

Catalase (CAT, E.C. 1.11.1.6) activity was measured with the method developed by Aebi [41], by evaluation of hydrogen peroxide decomposition, measured spectrophotometrically at the wavelength of 240 nm. One unit of CAT was defined as the amount of the enzyme which is needed to decompose one nmol of hydrogen peroxide within 1 minute. The results are presented as nmol H_2_O_2_/min/mg protein.

The activity of glutathione peroxidase (GPx, E.C. 1.11.1.9) was determined using Paglia and Valentine's method [42] based on the conversion of NADPH (reduced nicotinamide adenine dinucleotide) to NADP^+^ (nicotinamide adenine dinucleotide cation). The measurements were performed spectrophotometrically at 340 nm. One unit of GPx was represented as the amount of the enzyme necessary to catalyze the oxidation of 1 *μ*mol of NADPH within 1 minute [43]. The results are presented as mU/mg protein.

Glutathione reductase (GR, E.C. 1.8.1.7) activity was evaluated spectrophotometrically with the Mize and Langdon [44] method at the wavelength of 340 nm. It was assumed that one unit of GR catalyzing oxidation of 1 *μ*mol of NADPH within 1 minute. The results are presented as mU/mg protein.

The activity of superoxide dismutase (SOD, E.C. 1.15.1.1) was determined with the spectrophotometric method, according to Misra and Fredovich [45]. The absorbance changes accompanying adrenaline oxidation to adrenochrome were measured at the wavelength of 480 nm. One SOD unit corresponds to the amount of enzyme reducing adrenaline oxidation by 50%. The results are presented as mU/mg protein.

### 2.6. Nonenzymatic Antioxidant Barrier

The uric acid (UA) concentration was measured spectrophotometrically at the wavelength of 490 nm using the commercial kit (QuantiChromTMUric Acid Assay Kit DIUA-250; BioAssay Systems, Hayward, CA, USA) according to the manufacturer's instructions. The results are presented as *μ*mol/mg protein.

The concentration of reduced (GSH) and oxidized (GSSG) glutathione was evaluated colorimetrically. The determination was based on the enzymatic reaction between NADPH, DTNB (5,5′-Dithiobis-(2-nitrobenzoic acid), and GR. In order to determine GSSG concentration, the samples were incubated with 2-vinylpiridine to inhibit glutathione oxidation after neutralization with 1 M chlorhydrol triethanolamine to pH 6-7. The concentration of GSH was calculated as a difference in the levels of total glutathione and GSSG. The measurements were taken at the 412 nm wavelength [46, 47]. The results are presented as *μ*mol/mg protein.

Oxidation/reduction potential (redox ratio) was calculated based on the formula = [GSH]^2^/[GSSG] [48].

### 2.7. Antioxidant Status

Total antioxidant capacity (TAC) was determined by the Erel's method [49]. 2,2-Azinobis (3-ethylbenzene-thiazoline-6-sulfonate) (ABTS) was mixed with potassium persulfate and incubated at room temperature for 12 hours to obtain ABTS^+^. In the next step, 1 mL of ABTS^+^ was added to 10 *μ*L of samples, and the absorbance was read 735 nm wavelength. Results of decolorization were linear with increasing Trolox concentrations. The results are presented as *μ*mol/mg protein.

Total oxidant status (TOS) was evaluated colorimetrically by Erel's method [50], using the oxidation reaction of Fe^2+^ to Fe^3+^ ions. Fe^3+^ ions were then detected using xylenol orange. The results are presented as nmol H_2_O_2_ equivalent/mg protein.

The oxidative stress index (OSI) was calculated using the formula: OSI = [TOS, *μ*mol H_2_O_2_ equivalent/L[/[TAC, mmol Trolox/L] × 10 [51, 52].

### 2.8. Oxidative Stress

The concentration of thiobarbituric acid reactive substances (TBARS) was measured colorimetrically using thiobarbituric acid (TBA) method. 1,1,3,3-Tetraethoxypropane was used as the standard, and determination was performed at 535 nm [53, 54]. The results are presented as *μ*mol/mg protein.

The spectrophotometric detection evaluated the concentration of advanced oxidation protein products (AOPP). Potassium iodide and acetic acid were added to the wells, and the absorbance was read immediately at 340 nm [55, 56]. The results are presented as *μ*mol/mg protein.

Ischemia modified albumin (IMA) concentration was determined colorimetrically at 470 nm. The determination was based on the measurement of the exogenous cobalt (Co^2+^) binding facility of human plasma albumin [57, 58]. The results are presented as *μ*mol/mg protein.

### 2.9. Nitrosative Stress

Nitrate/nitrite (NOx) concentration was determined spectrofluorimetrically. Stable decomposition products of nitric oxide (NO) from the Griess reaction were evaluated by measuring absorbance at 543 nm wavelength [59]. The results are presented as *μ*mol/mg protein.

The peroxynitrite level was determined spectrophotometrically by measurement of the absorbance of nitrophenol at the wavelength of 320 nm. The nitrophenol production resulted from the decomposition of peroxynitrite followed by nitration of glycyltyrosine and 4-hydroxyphenyloacetic acid (4-HPA) [60, 61]. The results are presented as *μ*mol/mg protein.

3-Nitrotyrosine (3-NT) level was measured using the ELISA method. According to the manufacturer's instructions, a commercial kit (Nitrotyrosine ELISA; Immundiagnostik AG, Bensheim, Germany) was used. The results are presented as *μ*mol/mg protein.

S-Nitrosothiol concentration was measured spectrophotometrically based on the reaction of the Cu^2+^ ions with the Griess reagent [62]. The solution was shaken and incubated for 20 minutes, after which the absorbance was measured at 490 nm [63]. The results are presented as nmol/mg protein.

### 2.10. Inflammation and Lysosomal Function

Myeloperoxidase (MPO, EC 1.11.2.2) activity was analyzed spectrophotometrically using sulfanilamide hexadecyl trimethylammonium, ortho-dianisidinedihydrochloride, and hydrogen peroxide [37, 64]. The absorbance was measured at 450 nm. The results are presented as mU/mg protein.

The tumor necrosis factor-alpha (TNF-*α*) level was determined by the ELISA method using a commercially available kit (EIAab Science Inc. Wuhan; Wuhan, China) according to the manufacturer's instructions. The results are presented as pg/mL.

The activity of N-acetyl-*β*-hexosaminidase (HEX, EC 3.2.1.52) and *β*-glucuronidase (GLU, EC 3.2.1.31) was estimated colorimetrically at 405 nm using 4-nitrophenyl-N-acetyl-*β*-glucosaminide (HEX) and 4-nitrophenyl-*β*-D-glucuronide (GLU) as a substrate reaction [65, 66]. The results are presented as pKat/mg protein.

### 2.11. Statistical Analysis

Statistical analysis was performed using GraphPad Prism 8.4.3 for macOS (GraphPad Software, Inc. La Jolla, USA). The normality of the distribution was assessed using the Shapiro–Wilk test, while homogeneity of variance used the Levene test. For comparison of quantitative variables, the two-way analysis of variance (ANOVA) followed by the original FDR method of Benjamini and Hochberg was used. Multiplicity adjusted *p* value was also calculated. The relationship between the assessed biomarkers was evaluated using the Pearson correlation coefficient. The statistical significance level was set at *p* < 0.05.

The number of subjects was determined based on our previous experiment, assuming the test power = 0.8 and *α* = 0.05 (online *ClinCalc* sample size calculator). Erythrocyte GSH-Px, plasma GSH, TAC, TBARS, AOPP, and peroxynitrite were used for calculations, and the minimum number of subjects should be 13 (in one group).

## 3. Results

### 3.1. Exercise Performance

The duration of the GE under normoxic conditions was 20.2 ± 1.9 min, and the maximal work rate amounted to 5.1 ± 0.3 W/kg of body weight. The SpO_2_ was 98.0 ± 0.8 and 91.9 ± 3.0% at rest and at the end of the exercise, respectively. The heart rate at the point of exhaustion was 193 ± 8 bpm. In hypoxia, the average duration of the exercise was 18.0 ± 1.9 min, whereas the maximal work rate was 4.6 ± 0.4 W/kg. The SpO_2_ was 93.3 ± 3.4 and 84.3 ± 5.4% at rest and at the point of exhaustion, respectively. The heart rate at the end of the exercise was 190 ± 9 bpm.

The duration of the TT in normoxia was 61.1 ± 5.4 min, and the average work rate was 3.6 ± 0.3 W/kg of body weight. The SpO_2_ amounted to 97.8 ± 2.0 and 93.6 ± 2.3% at rest and at the end of the exercise, respectively. The average heart rate was 176 ± 9, 177 ± 8, and 179 ± 10 bpm after 10, 20, and 30 km of the TT, respectively. Under hypoxic conditions, the TT lasted 63.7 ± 3.0 min, whereas the average work rate was 3.3 ± 0.2 W/kg. The SpO_2_ was 93.3 ± 3.8 and 86.5 ± 2.7% at rest and at the end of the TT, respectively. The mean heart rate was 174 ± 11, 176 ± 13, and 180 ± 12 bpm after 10, 20, and 30 km of the TT, respectively.

### 3.2. Enzymatic Antioxidant Barrier

**Figure 1:**
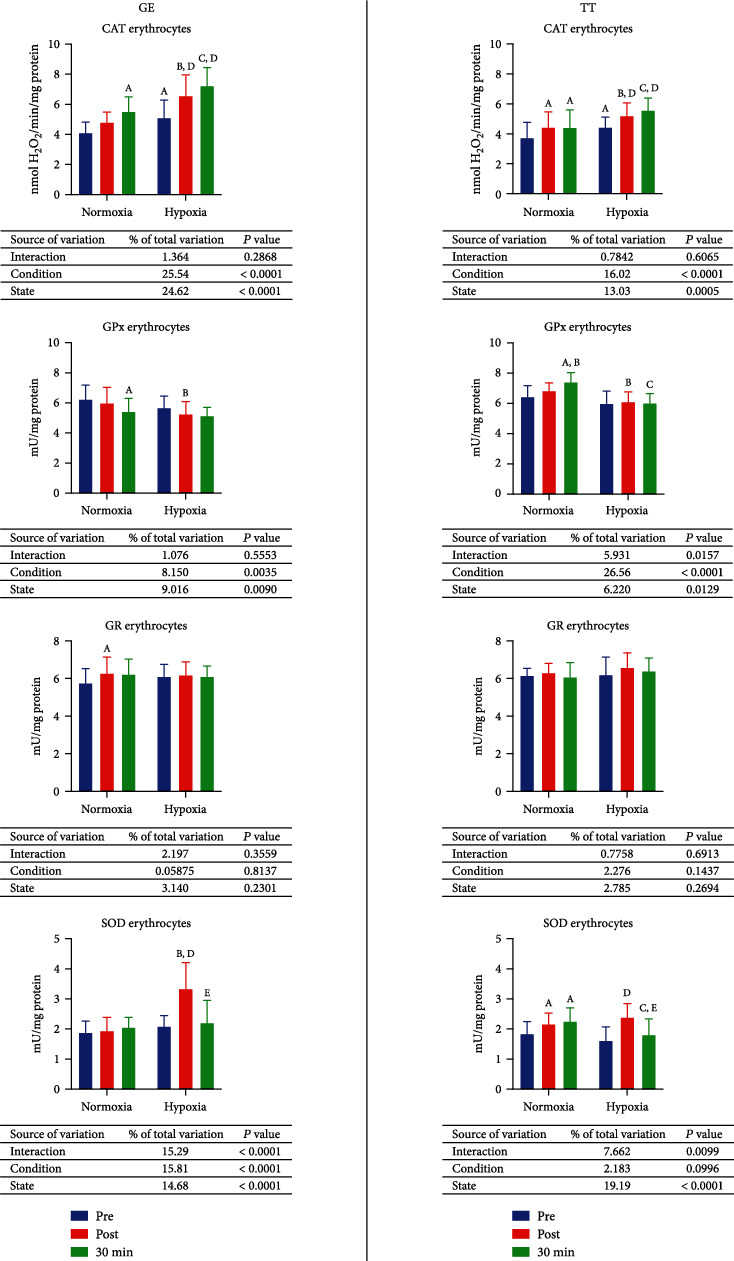
Effect of graded exercise until exhaustion (GE) and 30 km time trial (TT) on the enzymatic antioxidant barrier in normoxia and hypoxia. CAT: catalase; GPx: glutathione peroxidase; GR: glutathione reductase; SOD: superoxide dismutase. a, *p* < 0.05 vs. the value before exercise in normoxia; b, *p* < 0.05 vs. the value after the exercise in normoxia; c, *p* < 0.05 vs. the value after the exercise and 30 min of rest in normoxia; d, *p* < 0.05 vs. the value after the exercise in hypoxia; e, *p* < 0.05 vs. the value after the exercise in hypoxia; f, *p* < 0.05 vs. the value after the exercise 30 min of rest in hypoxia.

In normoxia, after the (GE) activity of glutathione reductase (GR) increased by 9% (*p* = 0.0464), while after 30 minutes of resting, the activity of catalase (CAT) rose 25.4% (*p* = 0.0003), and the activity of glutathione peroxidise (GPx) decreased by 13% (*p* = 0.0081), all comparing to the preexercise activity. In hypoxia, the postexercise activity of CAT and superoxide dismutase (SOD) was consecutively 28% (*p* = 0.0002) and 59.8% (*p* < 0.0001) greater than preexercise. After resting, the activity of CAT was 41.6% higher (*p* < 0.0001) than before the GE, and SOD activity was 33.9% lower (*p* < 0.0001) comparing to the evaluation performed immediately after exercise. When comparing the enzymatic activity in groups exercising hypoxia to those exercising normoxia, the CAT activity was 24% higher (*p* = 0.0088) before the GE. In postexercise measurement, the activity of CAT was 6% higher (*p* < 0.0001), the activity of GPx was 12.1% lower (*p* = 0.0175), and the activity of SOD was 71.7% greater (*p* < 0.0001). Moreover, the activity of CAT after resting was 41.6% higher (*p* < 0.0001) in hypoxia (Figure 1, GE).

After 30 km TT in normoxia, the activity of CAT and SOD after the exercise rose 18.8% (*p* = 0.0386) and 17.1% (*p* = 0.046) consecutively, and after resting for 30 minutes, the activity of GPx and SOD increased by 15.2% (*p* < 0.0001) and 22.3% (*p* = 0.01), respectively, all comparing to the activity before exercising. If compared to the postexercise value, after resting, the activity of GPx decreased by 24.4% (*p* = 0.0009). After the exercise in hypoxia, there was a significant increase in the activity of CAT (17.2%; *p* = 0.0242) and SOD (47.9%; *p* < 0.0001). Furthermore, the activity of SOD after resting was 24.4% lower (*p* = 0.0009) than after the exercise. Before TT, the activity of CAT in hypoxia was 18.9% (*p* < 0.0001) greater than in normoxia and 17.4% (*p* = 0.0233) greater after the exercise, while the postexercise SOD activity was 10.5% (*p* = 0.003) lower in hypoxia than in normoxia. Evaluation taken 30 minutes after the end of TT revealed that in hypoxia, the activity of GPx and SOD was significantly lower than in normoxia—18.7% (*p* < 0.0001) and 19.9% (*p* = 0.0051), respectively (Figure 1, TT).

### 3.3. Nonenzymatic Antioxidant Barrier

**Figure 2:**
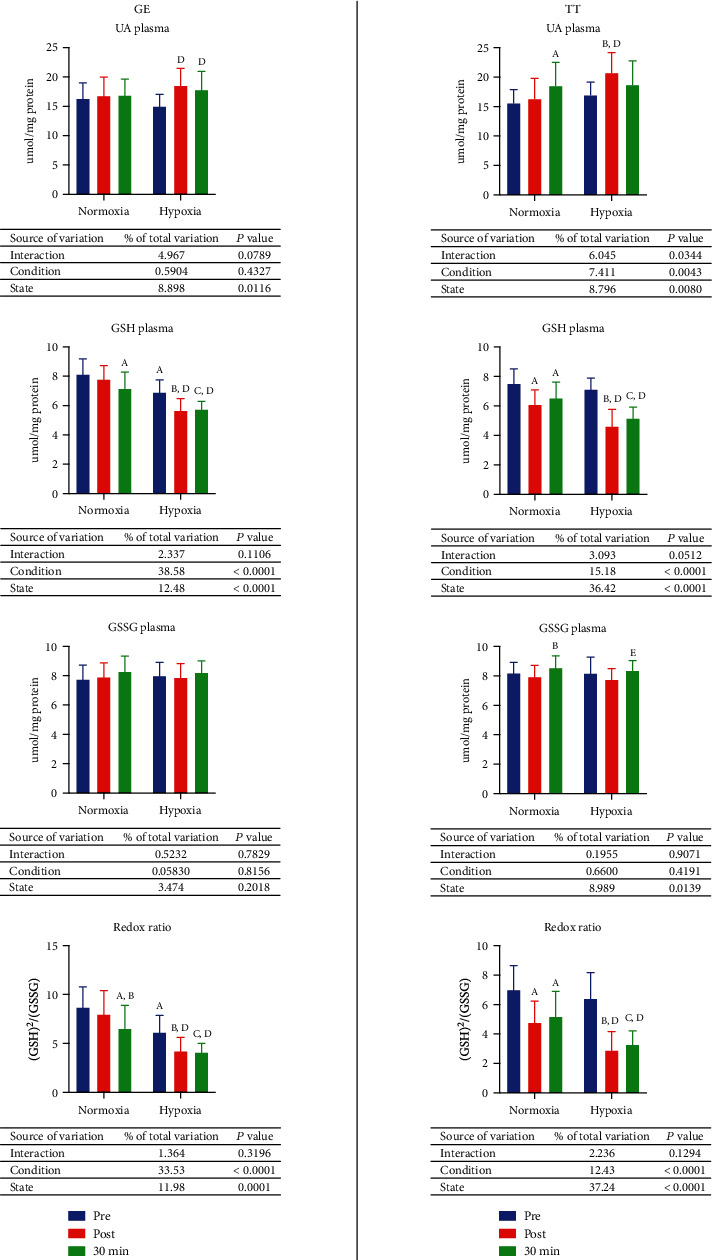
Effect of graded exercise until exhaustion (GE) and 30 km time trial (TT) on the nonenzymatic antioxidant barrier in normoxia and hypoxia. GSH: reduced glutathione; GSSG: oxidized glutathione; UA: uric acid. a, *p* < 0.05 vs. the value before exercise in normoxia; b, *p* < 0.05 vs. the value after the exercise in normoxia; c, *p* < 0.05 vs. the value after the exercise and 30 min of rest in normoxia; d, *p* < 0.05 vs. the value after the exercise in hypoxia; e, *p* < 0.05 vs. the value after the exercise in hypoxia; f, *p* < 0.05 vs. the value after the exercise 30 min of rest in hypoxia.

There were no significant changes in concentration of uric acid (UA), reduced (GSH), and oxidized (GSSG) glutathione or value of redox ratio in normoxia after GE compared to those values before exercise. However, after 30 minutes rest, the concentration of UA increased by 18.7% (*p* = 0.0058), while concentration of GSH and redox ratio lessened by 11.9% (*p* = 0.0031) and 24.8% (*p* = 0.0017) consecutively, comparing to preexercise values. Redox ratio decreased also in comparison to postexercise value (18%, *p* = 0.0343). In hypoxia, comparing to the measurements taken before exercise, immediately after GE, concentration of UA increased by 23.6% (*p* = 0.0006), and concentration of GSH and redox ratio decreased by 18% (*p* = 0.0002) and 31.1% (*p* = 0.0053) consecutively after the exercise. After 30 minutes of resting, redox ratio was 33.5% lower (*p* = 0.0028). Moreover, concentration of GSH and redox ratio was significantly lower in hypoxia than in normoxia both before (GSH 15%, *p* = 0.0002; redox ratio 29.3%, *p* < 0.0001) and after (GSH 30%, *p* < 0.0001; redox ratio 51.3%, *p* < 0.0001) GE, as well as after 30 minutes rest (GSH 19.7%, *p* < 0.0001; redox ratio 37.4%, *p* = 0.0004) (Figure 2, GE).

In normoxia, the concentration of GSH and redox ratio declined significantly after 30 km time trial—19% (*p* < 0.0001) and 31.6% (*p* < 0.0001), respectively. Thirty minutes after finishing the TT, UA concentration raised by 18.8% (*p* = 0.0143), and concentration of GSH (13%, *p* = 0.0048) and redox ratio (25.8%, *p* = 0.0009) decreased significantly compared to the values observed before conducting the time trial, while the GSSG concentration increased by 7.79% (*p* = 0.0366) comparing to the measurement taken immediately after the exercise. In hypoxia, 30 km time trial affected significantly the content of UA (increased by 22.3%, *p* = 0.0018), GSH (decreased by 35%, *p* < 0.0001), and redox ratio (decreased by 55%, *p* < 0.0001). After resting, there was a significant decrease in GSH concentration (27.5%, *p* < 0.0001) and redox ratio (48.8%, *p* < 0.0001) in comparison to those before TT, while GSSG content was 7.8% higher (*p* = 0.038) than right after the exercise. Moreover, postexercise content of UA was 27% greater (*p* = 0.0003) in hypoxia than in normoxia, while the concentration of GSH and redox ratio was significantly lower in hypoxia—24.2% (*p* < 0.0001) and 39.7% (*p* = 0.0005), respectively. After 30 minutes of resting, GSH content and redox ratio were 20.9% (*p* = 0.0001) and 36.8% (*p* < 0.0001) lower in hypoxia than in normoxia (Figure 2, TT).

### 3.4. Antioxidant Status

**Figure 3:**
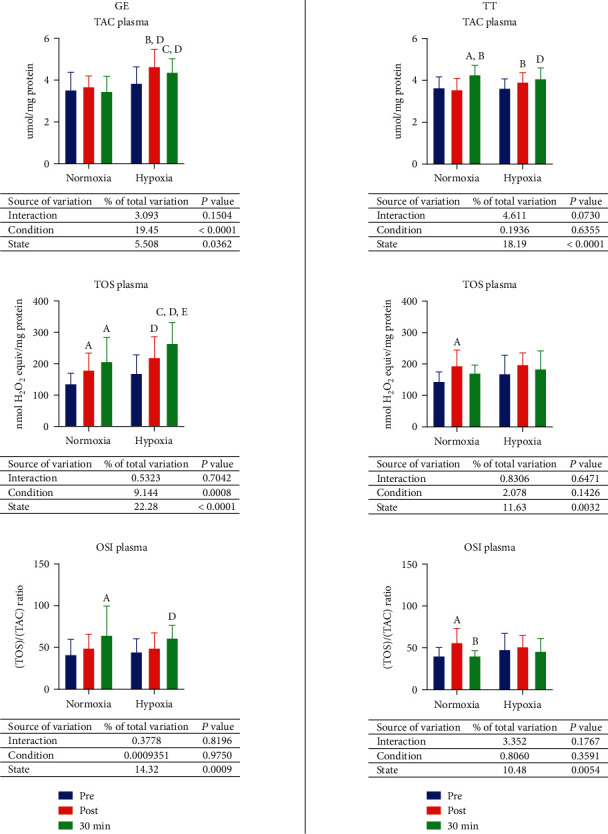
Effect of graded exercise until exhaustion (GE) and 30 km time trial (TT) on antioxidant status in normoxia and hypoxia. OSI: oxidative stress index; TAC: total antioxidant capacity; TOS: total oxidant status. a, *p* < 0.05 vs. the value before exercise in normoxia; b, *p* < 0.05 vs. the value after the exercise in normoxia; c, *p* < 0.05 vs. the value after the exercise and 30 min of rest in normoxia; d, *p* < 0.05 vs. the value after the exercise in hypoxia; e, *p* < 0.05 vs. the value after the exercise in hypoxia; f, *p* < 0.05 vs. the value after the exercise 30 min of rest in hypoxia.

Total oxidant status (TOS) was raised by graded exercise until exhaustion in normoxia by 31.9% (*p* = 0.0485), while total antioxidant capacity (TAC) and oxidative stress index (OSI) were not affected. Moreover, after 30 minutes of resting, TOS increased by 51.9% (*p* = 0.0016), and OSI increased by 55.8% (*p* = 0.0023) comparing to the preexercise. In hypoxia, both TAC and TOS were elevated after exercising—by 20.8% (*p* = 0.0025) and 29.7% (*p* = 0.0228), respectively. There were also significant rises of all described measurements after 30 minutes of rest in relation to the values observed before the exercise (TAC—13.6%, *p* = 0.044; TOS—56.5%, *p* < 0.0001; OSI—37.1%, *p* = 0.0276). TOS was also 20.6% higher (*p* = 0.0399) after resting than immediately after GE. Furthermore, postexercise TAC was 26.2% greater (*p* = 0.0003) in hypoxia than in normoxia, whereas both TAC and TOS values after resting were higher in hypoxia as well (26.4%, *p* = 0.0442; 28%, *p* = 0.009, respectively) (Figure 3, GE).

In normoxia, total antioxidant capacity was increased by 30 km time trial by 34.8% (*p* = 0.0023) while oxidative stress index was elevated by 39.7% (*p* = 0.0021). After resting, TAC was raised by 16.9% (*p* = 0.0006) compared to preexercise values and by 19.9% (*p* = 0.0001) compared to postexercise measurement. OSI was 28.4% (*p* = 0.0021) lower after resting than immediately after TT. Hypoxia did not affect postexercise markers' values, but resting for 30 minutes led to 12.6% growth (*p* = 0.0104) comparing to the preexercise analysis. There were no significant differences between preexercise values comparing normoxia and hypoxia, although postexercise TAC was 10.3% higher (*p* = 0.0392) in hypoxia than in normoxia (Figure 3, TT).

### 3.5. Oxidative Stress

**Figure 4:**
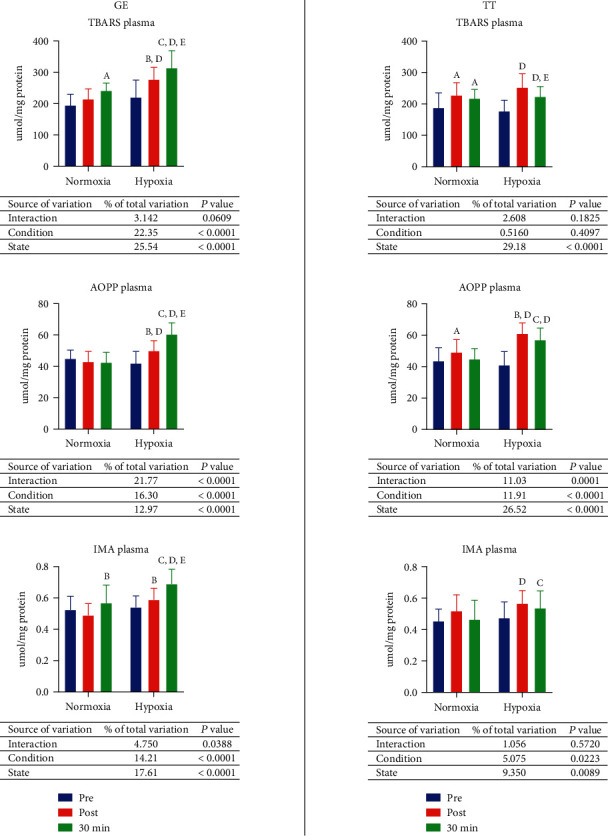
Effect on graded exercise until exhaustion (GE) and 30 km time trial (TT) on oxidative stress in normoxia and hypoxia. AOPP: advanced oxidation protein products; IMA: ischemia modified albumin; TBARS: thiobarbituric acid reactive substances. a, *p* < 0.05 vs. the value before exercise in normoxia; b, *p* < 0.05 vs. the value after the exercise in normoxia; c, *p* < 0.05 vs. the value after the exercise and 30 min of rest in normoxia; d, *p* < 0.05 vs. the value after the exercise in hypoxia; e, *p* < 0.05 vs. the value after the exercise in hypoxia; f, *p* < 0.05 vs. the value after the exercise 30 min of rest in hypoxia.

Graded exercise until exhaustion in normoxia did not influence oxidative stress marker concentration assessed directly after exercise. However, there were significant changes in TBARS (24% increase compared to preexercise value, *p* = 0.0017) and ischemia modified protein (IMA) (15.9% increase compared to postexercise value, *p* = 0.0121) contents when measured after 30 minutes of rest. In hypoxia, TBARS concentration was elevated by 25.8% (*p* = 0.0002), and advanced oxidation protein product (AOPP) content was increased by 18.8% (*p* < 0.0001) immediately after exercise. After the rest, the significant increase of the concentration of all evaluated substances was observed—TBARS by 42.2% (*p* < 0.0001), AOPP by 43.8% (*p* < 0.0001), and IMA by 27.6% (*p* < 0.0001) comparing to the values before GE and TBARS by 13% (0.0141), AOPP by 21% (*p* < 0.0001), and IMA by 17.2% (*p* = 0.0003) comparing to the values after GE. There were also no differences between preexercise concentrations in normoxia and hypoxia, while contents of all described substances were significantly higher in hypoxia than in normoxia after the GE—TBARS 29.4% (*p* < 0.0001), AOPP 16.5% (*p* = 0.0032), and IMA 20.1% (*p* = 0.0016)—and after 30 minutes of resting—TBARS 29.7% (*p* < 0.0001), AOPP 21% (*p* < 0.0001), and IMA 21.5% (*p* = 0.0001) (Figure 4, GE).

After 30 km time trial in normoxia, the concentration of TBARS was increased by 21.5% (*p* = 0.0034), and the content of AOPP was 12.9% (*p* = 0.0401) higher. After 30 minutes of rest, the TBARS concentration was elevated by 16.1% (*p* = 0.0267). In hypoxia, all postexercise oxidative stress markers' concentrations were elevated—TBARS by 42.7% (*p* < 0.0001), AOPP by 49% (*p* < 0.0001), and IMA by 19.4% (*p* = 0.0101). Directly after the exercise, but after 30 minutes, the content of TBARS decreased by 11.5% (*p* = 0.0328). There were no differences between preexercise values in hypoxia and normoxia. The concentration of AOPP after TT in hypoxia was 24.2% greater (*p* < 0.0001) than in normoxia. After the rest, the contents of AOPP and IMA were also higher in hypoxia—39% (*p* < 0.0001) and 15.7% (*p* = 0.0407) consecutively (Figure 4, TT).

### 3.6. Nitrosative Stress

**Figure 5:**
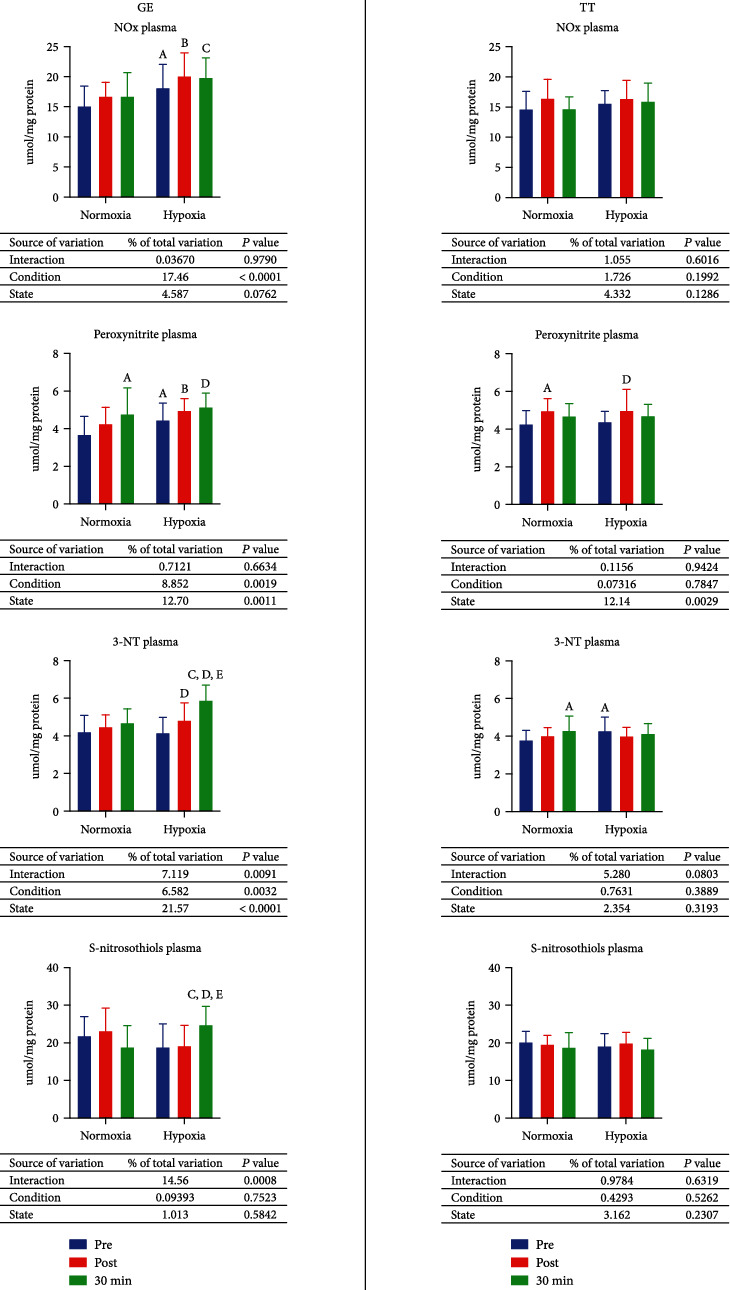
Effect on graded exercise until exhaustion (GE) and 30 km time trial (TT) on nitrosative stress in normoxia and hypoxia. 3-NT: 3-nitrotyrosine; NO_x_: nitrate/nitrite. a, *p* < 0.05 vs. the value before exercise in normoxia; b, *p* < 0.05 vs. the value after the exercise in normoxia; c, *p* < 0.05 vs. the value after the exercise and 30 min of rest in normoxia; d, *p* < 0.05 vs. the value after the exercise in hypoxia; e, *p* < 0.05 vs. the value after the exercise in hypoxia; f, *p* < 0.05 vs. the value after the exercise 30 min of rest in hypoxia.

Besides 29.8% increase (*p* = 0.0014) in peroxynitrite level after 30 minutes of resting in relation to the pre-GE value and 18.8% decrease (*p* = 0.0291) in S-nitrosothiol concentration after the rest compared to the measurement conducted directly after exercise, there was no other influence of GE in normoxia on nitrosative stress markers. In hypoxia, the 3-nitrotyrosine (3-NT) level was elevated by 15.9% (*p* = 0.0223) after the exercise. After resting, levels of peroxynitrite and 3-NT and concentration of S-nitrosothiols increased by 15.6% (*p* = 0.0397), 41.1% (*p* < 0.0001), and 31.2% (*p* = 0.0034), respectively, in comparison to the preexercise evaluation. Moreover, 3-NT level and S-nitrosothiol content were greater after 30 minutes of resting than immediately after exercise (21.8%, *p* = 0.0004; 29%, *p* = 0.0055 consecutively). Comparing the group exercising in hypoxia to the group exercising in normoxia—in hypoxia, before exercise, the content of NO_x_ was 20.2% higher (*p* = 0.0144), and the level of peroxynitrite was 21.1% greater (*p* = 0.0219). In comparison, after exercise, the NO_x_ concentration and peroxynitrite level were 20.3% (*p* = 0.007) and 16.4% (*p* = 0.0393) higher, and S-nitrosothiol concentration was 17.3% lower (*p* = 0.044). Furthermore, 30 minutes after finishing the exercise, NO_x_ concentration, 3-NT level, and S-nitrosothiol content were also significantly higher in hypoxia—18.5% (*p* = 0.0133), 25.4% (*p* < 0.0001), and 31.4% (*p* = 0.0055), respectively (Figure 5, GE).

After a 30 km TT in normoxia, the only significant change was 16.4% (*p* = 0.0083) increase in peroxynitrite level. In hypoxia, the level of peroxynitrite rose 13.5% (*p* = 0.0252) after the exercise, while the level of 3-NT increased by 13.1% (*p* = 0.0193) after 30 minutes of rest, both comparing to the preexercise levels. Moreover, before the exercise, the concentration of 3-NT was 12.7% higher (*p* = 0.0228) in hypoxia than normoxia. No other significant changes were observed (Figure 5, TT).

### 3.7. Inflammation and Lysosomal Function

**Figure 6:**
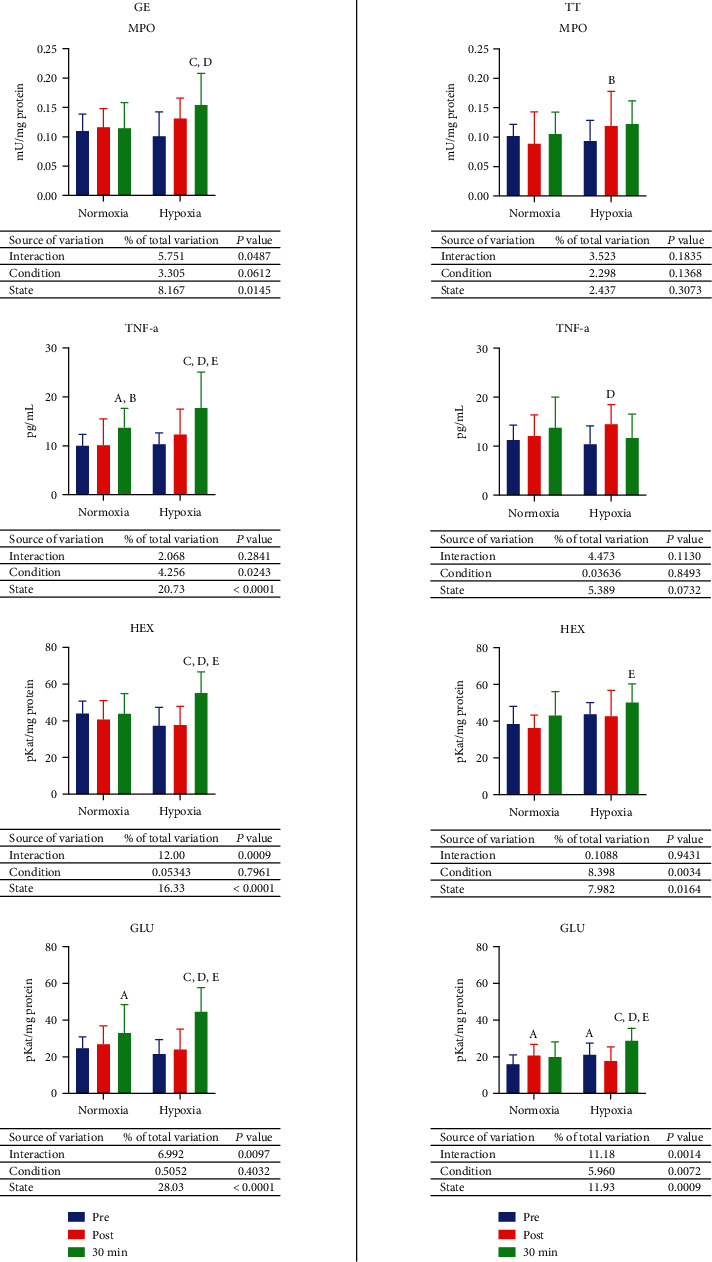
Effect on graded exercise until exhaustion (GE) and 30 km time trial (TT) on inflammation and lysosomal function in normoxia and hypoxia. GLU: *β*-glucuronidase; HEX: N-acetyl-*β*-hexosaminidase; MPO: myeloperoxidase; TNF-*α*: tumor necrosis factor-alpha. a, *p* < 0.05 vs. the value before exercise in normoxia; b, *p* < 0.05 vs. the value after the exercise in normoxia; c, *p* < 0.05 vs. the value after the exercise and 30 min of rest in normoxia; d, *p* < 0.05 vs. the value after the exercise in hypoxia; e, *p* < 0.05 vs. the value after the exercise in hypoxia; f, *p* < 0.05 vs. the value after the exercise 30 min of rest in hypoxia.

After graded exercise until exhaustion in normoxia, there were no significant changes in the activity of myeloperoxidase (MPO), level of tumor necrosis factor-alpha (TNF-*α*), or the activity of N-acetyl-*β*-hexosaminidase (HEX) and *β*-glucuronidase (GLU). After 30 minutes of resting, level of TNF-*α* and the activity of GLU increased by 36.6% (*p* = 0.0279) and 33.3% (*p* = 0.0322) comparing to preexercise, and the level of TNF-*α* rose 35% (*p* = 0.033) comparing to postexercise value as well. In hypoxia, the activity of all examined enzymes, as well as the level of TNF-*α*, increased after resting comparing to the measurement taken before exercise (MPO—52.4%, *p* = 0.0002; TNF-*α*—71%, *p* < 0.0001; HEX—47.7%, *p* < 0.0001; GLU—105.4%, *p* < 0.0001). The level of TNF-*α* and activity of HEX and GLU were significantly higher after rest also comparing to the assessment performed directly after exercise—43.9% (*p* = 0.0014), 47.7% (*p* < 0.0001), and 84.7% (*p* < 0.0001), respectively. No significant differences between inflammation and lysosomal function markers between values in normoxia and hypoxia were observed in measurements performed before and after GE. However, after 30 minutes, activity of all investigated enzymes and level of TNF-*α* were higher in hypoxia than in normoxia—MPO 34.4% (*p* = 0.005), TNF-*α* 28.3% (*p* = 0.0161), HEX 46% (*p* < 0.0001), and GLU 34.9% (*p* = 0.0031) (Figure 6, GE).

After 30 km TT in normoxia, the activity of GLU increased by 29.8% (*p* = 0.038) considering pre-TT values. In hypoxia, the level of TNF-*α* raised by 38.3% (*p* = 0.0109) subsequently to the exercise, while after 30 minutes, the activity of GLU raised by 6.4% (*p* = 0.0012) comparing to the activity before exercise and by 9% (*p* < 0.0001) comparing to the activity directly after exercise, and the activity of HEX increased by 17.2% (*p* = 0.043) in relation to postexercise measurement. Comparing exercising in hypoxia to exercising in normoxia, the preexercise GLU activity in hypoxia was 31.7% (*p* = 0.0279) higher, while the postexercise activity of MPO was 33.6% (*p* = 0.454) greater too. After resting, the activity of GLU was also 7.2% higher (*p* = 0.0002) in hypoxia (Figure 6, TT).

### 3.8. Correlations

**Figure 7:**
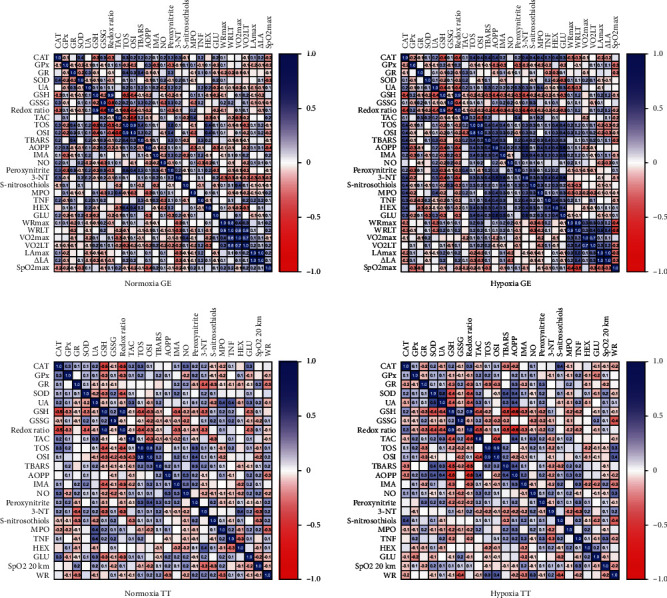
Correlation heat maps between redox and exercise parameters.

The results of correlation analysis are shown in Figure 7. Of particular note is the negative correlation between GSH and TOS levels (*r* = −0.465; *p* = 0.001) and the positive correlation between peroxynitrite and TOS (*r* = 0.458; *p* = 0.001) in the GE group in normoxic conditions. In the GE group in hypoxia, AOPP levels correlated positively with 3-NT content (*r* = 0.571; *p* < 0.0001) and GLU activity (*r* = 0.62; *p* < 0.0001), whereas IMA levels correlated with 3-NT (*r* = 0.55; *p* < 0.0001) and GLU (*r* = 0.487; *p* < 0.0001). Interestingly, UA concentration was also correlated negatively with WRmax (*r* = −0.326; *p* = 0.029). In the TT group in normoxia, GSH concentration (*r* = −0.496; *p* < 0.0001) and redox ratio (*r* = −0.496; *p* = 0.001) correlated negatively with CAT activity, whereas in hypoxia, UA concentration correlated positively with SOD activity (*r* = 0.487; *p* = 0.013), and GSH correlated negatively with TBARS (*r* = −0.486; *p* < 0.0001) and AOPP level (*r* = −0.605; *p* = 0.0001). Similarly, redox ratio correlated negatively with TAC (*r* = −0.451; *p* = 0.001), TBARS (*r* = −0.468; *p* = 0.001), and AOPP (*r* = −0.615; *p* < 0.0001) concentrations (Figure 7).

## 4. Discussion

Our study is the first to evaluate the effect of different acute exercise protocols performed under normoxia and hypoxia on antioxidant status, oxidative and nitrosative damage, inflammation, and lysosomal function. We have shown that both graded exercise until exhaustion (GE) and 30 km time trial (TT) impair the efficiency of antioxidant systems and induce oxidative and nitrosative stress, with hypoxia causing more significant disruption in redox homeostasis and inflammation.

In recent years, there has been a marked increase in interest in mountain sports [67]. Apart from its undoubted advantages, this type of activity is not without health risks. Limited oxygen diffusion through the pulmonary capillaries contributes to tissue hypoxia and the overproduction of free radicals [32, 67]. ROS sources under these conditions include primarily reduced partial pressure of oxygen in the air (hypobaric hypoxia), intense physical activity, and auto-oxidation of catecholamines. Although various adaptive mechanisms can partially compensate for tissue hypoxia (e.g., hyperventilation, tachycardia, increased cardiac output, and enhanced hemoglobin and erythrocytes content), the most effective blood response does not appear until several days later [32].

The present study generally demonstrated the strengthening of enzymatic antioxidant systems during hypoxic exercise (GE: ↑ CAT, ↑ SOD; TT: ↑ CAT vs. normoxia). Changes in the enzymatic antioxidant barrier may reflect various functional/pathophysiological states. The initial increase in enzyme activity is usually adaptation to higher production of ROS and RNS, whereas the subsequent decrease results from depletion of the antioxidant reserves. Of particular note are the erythrocyte enzymes (GPx and CAT) that degrade hydrogen peroxide. GPx plays a key role in H_2_O_2_ degradation at physiological concentrations by reducing hydrogen peroxide with the simultaneous conversion of GSH to its oxidized form (GSSG). However, under H_2_O_2_ overproduction, CAT exhibits greater enzymatic activity as evidenced by the Michaelis–Menten constant (Km) for GPx (1 × 10^−6^ M) and CAT (2.4 × 10^−4^ M) [68, 69]. Although we did not directly assess the rate of free radical formation, the increase in CAT and decrease in GPx activity (versus normoxia) indicate a higher intensity of oxidative processes during hypoxic exercise. Enhanced plasma TOS in hypoxia also supports this hypothesis. It is well known that TOS determines the total amount of oxidants in a biological system [50]. Considering that free radicals can mutually enhance their production, TOS provides more information than evaluation of individual ROS/RNS separately. However, what could constitute an additional source of free radicals in hypoxic exercise? During tissue hypoxia (as during tissue ischemia), xanthine dehydrogenase is converted to XO, donating an electron to molecular oxygen. The reaction catalyzed by XO produces superoxide anions and hydrogen peroxide [2, 30, 31, 70], explaining the increase in SOD and CAT activity under hypoxic exercise. However, overactivation of nitric oxide synthases (NOS), especially inducible NOS (iNOS), also occurs in these conditions [71, 72]. Excess nitric oxide (NO) concentrations inhibit cytochrome oxidase activity, which in turn intensifies O_2_^-•^ production [73, 74]. If the full O_2_ supply is restored, there is an increased formation of ROS referred to as the “oxygen paradox” [75, 76]. Therefore, enhanced CAT activity observed in our study is not surprising (increase at each time interval versus normoxic exercise). Interestingly, the activity of antioxidant enzymes (CAT, SOD) and total oxidative capacity (TOS, OSI) were also relatively higher in high-intensity exercise until exhaustion (GE). If O_2_ supply to the cells is insufficient, energy is produced in the low-efficiency process of anaerobic glycolysis, leading to an increase in H^+^ ions and lactate concentrations. The consequence is the loss of ability to maintain ionic homeostasis, particularly an increase in the extracellular concentration of K^+^ and the accumulation of Na^+^ and Ca^2+^, which is responsible for ROS overproduction [77, 78].

The signaling effects of hydrogen peroxide are associated with proteins recording changes in cellular redox status. The molecules responsible for transmitting the H_2_O_2_ signal to the nucleus are low molecular weight thiols, of which reduced glutathione is an essential intracellular source [79, 80]. Therefore, it is not surprising that GSH concentrations were significantly lower in athletes exercising in hypoxia compared to normoxia. Since GSSG concentrations and GR activity were unchanged, the decrease in GSH concentration may be due to the oxidation of enzymes responsible for glutathione synthesis or the formation of S-conjugates with glutathione and proteins. In addition to its antioxidant role, GSH participates in DNA replication and apoptosis and regulates the thiol groups of proteins in their reduced state [81, 82]. Therefore, maintenance of adequate GSH levels is crucial for proper cellular function. In our study, despite strengthening the antioxidant barrier under hypoxia, there was a redox imbalance in favor of oxidative reactions (GE: ↑ TOS, ↓ redox ratio). This results in enhanced oxidation of plasma proteins (GE: ↑ AOPP, ↑ IMA; TT: ↑ AOPP) and lipids (GE: ↑ TBARS), which indicates the occurrence of systemic oxidative stress. This may be confirmed by the negative correlation between GSH concentration and TBARS and AOPP and between redox ratio and TAC, TBARS, and AOPP. Of particular note is the increase in IMA levels during hypoxic exercise. IMA is the earliest biomarker of tissue ischemia, whereas decreased oxygen saturation, ischemic reperfusion, acidosis, sodium/calcium pump dysfunction, and higher oxidative stress are factors causing conformational changes of albumin [57]. The increase in total antioxidant capacity may also be controversial (GE: ↑ TAC both after exercise and hypoxia vs. normoxia). Nevertheless, 70-80% of plasma TAC represents nonenzymatic uric acid (UA), with a robust prooxidant effect in high concentrations [83, 84]. UA can generate free radicals by reacting with peroxynitrite or forming alkylated proteins, lipids, and carbohydrates [85]. Higher UA concentrations were observed in previous studies after one-time and regular high-intensity physical training [2, 20, 86–88]. UA is the end product of purine catabolism formed in a XO-catalyzed reaction from xanthine. Under hypoxic/ischemic conditions, hypoxanthine formed from ATP decomposition is accumulated in the cell and then metabolized to xanthine with the generation of ROS/RNS upon reperfusion [89, 90].

The H_2_O_2_ production may also be affected by nitric oxide metabolism [91, 92]. In our study, higher NO_x_ bioavailability with a concomitant increase in CAT activity could be explained by intensified peroxynitrite (ONOO^−^) formation influenced by an acute hypoxic intervention. Indeed, the superoxide radicals formed in the XO-catalyzed pathway react with NO to generate the highly reactive ONOO^−^ [92]. Peroxynitrite is a powerful oxidizing and nitrating agent that initiates lipid peroxidation and oxidation of thiols/aromatic amino acids with an efficiency of at least 1000-fold higher than hydrogen peroxide [93, 94]. Tyrosine residues are particularly sensitive to ONOO^−^ damage; hence, the increase in 3-NT concentrations (both after exercise and in hypoxic conditions) is not surprising. Interestingly, peroxynitrite formation occurs typically under increased systemic inflammation [93, 94]. This may be supported by the results of our study (GE: ↑ NO, ↑ ONOO^−^, ↑ MPO, ↑ TNF-*α*). Of particular note is higher MPO activity after hypoxic exercise. Indeed, MPO is released by neutrophils and monocytes during inflammatory cell activation [95]. It is involved in hypochlorous acid production, which exacerbates oxidative stress and initiates acute inflammation [95, 96]. It is well known that higher secretion of cytokines, chemokines, and growth factors is a physiological response to decreased arterial blood O_2_ saturation and microdamage of muscle fibers. Activated neutrophils and macrophages can remove fragments of damaged muscle tissue induced by NO and H_2_O_2_ signaling [97, 98]. Simultaneously, IL-1, IL-2, IL-6, and TNF-*α* may stimulate white blood cells to produce significant amounts of NO through prolonged iNOS activation [99, 100]. In these conditions, XO and NOX are also induced, which, by positive feedback, enhances nitrosative cell injury (GE: ↑ NO_x_, ↑ ONOO^−^, ↑ 3-NT). A consequence of enhanced inflammatory response and oxidative/nitrosative stress can be damage to the lysosomal membrane and the release of lysosomal enzymes into the circulation (GE: ↑ HEX, ↑ GLU). Interestingly, lysosomal dysfunction, mitochondrial energy metabolism, and impaired ion homeostasis are essential sources of ROS during physical exercise [101–103]. However, NO signaling activity may also depend on protein S-nitrosylation, as evidenced by increased S-nitrosothiols under hypoxic conditions. It is well known that NO-mediated protein S-nitrosylation plays a vital role in the adaptation to endurance exercise/hypoxia by increasing the PGC-1*α* (peroxisome proliferator-activated receptor gamma coactivator 1-alpha) expression [104, 105]. Nevertheless, enhanced S-nitrosylation can also end in the formation of protein disulfide and a nitroxyl residue, which irreversibly alters the biological properties of proteins.

Physical exercise is indicated in both health and disease. Although our study does not explain it, individuals with diseases with oxidative stress etiology (e.g., metabolic, neurodegenerative, and immune diseases) should be cautious during acute hypoxic training. This may exacerbate disturbances in redox homeostasis and inflammation. Antioxidant supplementation during acute hypoxic exercise also remains an open question.

Unfortunately, our work has numerous limitations. These include the relatively small number of participants and the evaluation of only selected biomarkers of oxidative stress, inflammation, and lysosomal function. Our study also does not explain the molecular mechanisms responsible for the observed redox disturbances. Research on nonprofessional athletes is also essential.

To summarize, our study shows that even a single session of physical exercise disrupts the enzymatic and nonenzymatic antioxidant barrier leading to enhanced oxidative and nitrosative damage at a systemic level. High-intensity exercise of short duration alters redox homeostasis more than prolonged aerobic exercise, while hypoxia significantly exacerbates oxidative stress, inflammation, and lysosomal dysfunction in athletic subjects. Although we have reported the most commonly assessed circulating redox biomarkers, further studies are needed to elucidate the molecular basis of the observed relationships. Studies on larger groups of athletes are also advisable.

## Data Availability

The datasets generated for this study are available on request to the corresponding author.
